# The Protective Effect of Alpha-Mangostin against Cisplatin-Induced Cell Death in LLC-PK1 Cells is Associated to Mitochondrial Function Preservation

**DOI:** 10.3390/antiox8050133

**Published:** 2019-05-15

**Authors:** Laura María Reyes-Fermín, Sabino Hazael Avila-Rojas, Omar Emiliano Aparicio-Trejo, Edilia Tapia, Isabel Rivero, José Pedraza-Chaverri

**Affiliations:** 1 Department of Biology, Faculty of Chemistry, National Autonomous University of Mexico (UNAM), Mexico City 04510, Mexico; laura_refe@yahoo.com.mx (L.M.R.-F.); shaggy_goose@hotmail.com (S.H.A.-R.); emilianoaparicio91@gmail.com (O.E.A.-T.); 2 Department of Nephrology and Laboratory of Renal Pathophysiology, National Institute of Cardiology “Ignacio Chávez”, Mexico City 14080, Mexico; ediliatapia@hotmail.com; 3 Department of Pharmacy, Faculty of Chemistry, UNAM, Mexico City 04510, Mexico; riveroic@yahoo.com.mx

**Keywords:** alpha-mangostin, cisplatin, nephrotoxicity, mitochondria function, mitophagy

## Abstract

Cis-dichlorodiammineplatinum II (CDDP) is a chemotherapeutic agent that induces nephrotoxicity by different mechanisms, including oxidative stress, mitochondrial dysfunction, autophagy, and endoplasmic reticulum stress. This study aimed to evaluate if the protective effects of the antioxidant alpha-mangostin (αM) in CDDP-induced damage in proximal tubule Lilly laboratory culture porcine kidney (LLC-PK1) cells, are related to mitochondrial function preservation. It was found that αM co-incubation prevented CDDP-induced cell death. Furthermore, αM prevented the CDDP-induced decrease in cell respiratory states, in the maximum capacity of the electron transfer system (E) and in the respiration associated to oxidative phosphorylation (OXPHOS). CDDP also decreased the protein levels of voltage dependence anion channel (VDAC) and mitochondrial complex subunits, which together with the reduction in E, the mitofusin 2 decrease and the mitochondrial network fragmentation observed by MitoTracker Green, suggest the mitochondrial morphology alteration and the decrease in mitochondrial mass induced by CDDP. CDDP also induced the reduction in mitochondrial biogenesis observed by transcription factor A, mitochondria (TFAM) decreased protein-level and the increase in mitophagy. All these changes were prevented by αM. Taken together, our results imply that αM’s protective effects in CDDP-induced toxicity in LLC-PK1 cells are associated to mitochondrial function preservation.

## 1. Introduction

Cis-dichlorodiammineplatinum II (CDDP) is a platinum compound frequently used in many types of cancers [1,2]. However, the main side-effect of CDDP is its nephrotoxicity, especially in proximal tubule epithelial cells. It is estimated that 20 to 30% of patients treated with CDDP developed transient episodes of acute kidney injury, which can progress to chronic kidney disease, depending on the dose and individual pharmacokinetics [2]. The CDDP-toxicity mechanisms involve DNA damage, oxidative stress, mitochondrial damage, endoplasmic reticulum (ER) stress, autophagy, and apoptotic cell death [3,4]. 

In recent years, the use of biomolecules with antioxidant activity has been widely studied to mitigate the nephrotoxic effects induced by CDDP [5]. Alpha-mangostin (αM), a bioactive compound with direct antioxidant properties that can be extracted from the *Garcinia mangostana* tree, has been broadly used in Asian traditional medicine and its nephroprotective effect has been demonstrated experimentally [6,7]. αM is a prenylated xanthone with antioxidant, anti-inflammatory, and anti-apoptotic properties [8,9]. Additionally, our research group demonstrated αM’s protective effects in CDDP-induced nephrotoxicity, related to the prevention of oxidant and nitrate stress increase, glutathione decrease, tumor necrosis factor alpha (TNF-α) and p53 increase, and apoptosis induction [3,7,9]. Recently, the modulation of mitochondrial function, autophagy, and ER stress have been related to αM’s protective effects in cancer and diabetic nephropathy models [10,11,12]. However, the participation of these processes in αM’s protection in CDDP-induced nephrotoxicity has not been studied yet.

Mitochondria are double membrane organelles, which regulate important functions related to energetic homeostasis and cell death. Additionally, mitochondria are considered as one of the main reactive oxygen species (ROS) producers in the cell [13]. It is a widely accepted concept that mitochondrial damage is one of the principal mechanisms involved in CDDP-induced nephrotoxicity. CDDP induces mitochondrial membrane potential loss, as well as alterations in bioenergetics, dynamics (balance between fusion and fission), biogenesis and mitophagy, which favor the induction of apoptosis [14,15,16,17]. Additionally, the higher dependence of mitochondrial adenosine triphosphate (ATP) production in the proximal tubule compared with other nephron segments, make the proximal tubule more susceptible to CDDP-damage [2,13].

On the other hand, autophagy is a multistep pathway that degrades and recycles damaged macromolecules and organelles, to maintain intracellular homeostasis. This process involves the sequestration of damaged components inside a double membrane vesicle (autophagosome) and their subsequent degradation when the autophagosome fuses with the lysosome (autolysosome) [18]. Although autophagosome and autolysosome formation involves multiple protein complexes and multiple steps, the increase of the lipidated microtubule-associated protein 1 light chain 3 alpha (LC3) form (commonly refer as LC3-II) and the decrease of p62 levels have been widely used to evaluate the induction of autophagy [19]. In the CDDP nephrotoxicity model, it has been suggested that autophagy induction acts as a protective mechanism early on [20,21,22]. Recent studies also show that mitophagy (a mitochondrial-specific type of autophagy) has a protective role in CDDP nephrotoxicity [22,23]. Under mitochondrial damage or depolarization, the induction of mitophagy helps to maintain the mitochondrial quality control and, therefore, the cellular homeostasis [19]. In CDDP-induced nephrotoxicity, the mitophagy clearance of damaged mitochondria mediated by the phosphatase and tensin homologue (PTEN) induced kinase 1/parkin RBR E3 ubiquitin protein ligase (PINK1/Parkin) pathway has shown protective effects [22,24].

This study aimed to evaluate if the protective effects of αM in CCDP-induced damage in Lilly laboratory culture porcine kidney (LLC-PK1) cells, was related to αM regulation of mitochondrial function and autophagy (especially mitophagy).

## 2. Materials and Methods 

### 2.1. Reagents

Alpha-mangostin (αM) was obtained as described in our previous studies [25] from Garcinia mangostana (DNP International Inc., Whittier, CA, USA) with 98% purity. The LLC-PK1 (ATTC^®^ CL-101) epithelial cell line, derived from proximal tubular cells of the porcine kidney, was acquired from American Type Culture Collection (ATCC, Rockville, MD, USA). Cis-dichlorodiammineplatinum II (CDDP), trypan blue, fluorescein diacetate (FDA), tris(hydroxymethyl)aminomethane, sodium deoxycholate, 4-nonylphenyl-polyethylene glycol (NP-40), sodium fluoride (NaF), D-(+)-glucose, sodium pyrophosphate (Na_4_P_2_O_7_), sodium orthovanadate (Na_3_VO_4_), Triton X-100, glycerophosphate, 4-(2-hydroxyethyl)piperazine-1-ethanesulfonic acid (HEPES), Tween 20, glycerol, ethylenediaminetetraacetic acid (EDTA), dimethyl sulfoxide (DMSO), antimycin A, ethylene glycol-bis(2-aminoethylether)-N,N,N′,N′-tetraacetic acid (EGTA), dinitrophenol (DNP), magnesium chloride (MgCl_2_) tetrahydrate, oligomycin, rotenone, sodium L-ascorbate, sodium azide (NaN3) and tetramethyl-p-phenylene diamine (TMPD), sodium phosphate monobasic (NaH_2_PO_4_), sodium phosphate dibasic (Na_2_HPO_4_), sodium dodecyl sulfate (SDS), ponceau S, chloroquine diphosphate salt (CQ), wortmannin and antibodies against microtubule-associated protein light chain 3B (LC3, L7543), ubiquitin-binding protein p62 (p62, P0067) and α-tubulin (α-Tub, T9026) were purchased from Sigma Aldrich (St. Louis, MO, USA). Dulbecco’s Modified Eagle’s Medium (DMEM), fetal bovine serum (FBS), and penicillin/streptomycin were purchased from Biowest (Riverside, MO, USA). Calcium chloride (CaCl_2_), sodium bicarbonate (NaHCO_3_), sodium chloride (NaCl), and hydrochloric acid (HCl) were purchased from J.T. Baker (Xalostoc, Edo. Mex, Mexico). Potassium chloride (KCl) was purchased from Mallinckrodt plc. (St. Louis, MO, USA). Protease inhibitor cocktail was purchased from Roche Applied Science (Mannheim, Germany). MitoTrackerTM Green FM was purchased from Thermo Fisher Scientific. Antibodies against malondialdehyde (MDA, ab6463), peroxisome proliferator-activated receptor gamma (PPARγ) coactivator 1α (PGC-1α, ab54481), 4-hydroxynonenal (4HNE, ab5605), parkin RBR E3 ubiquitin protein ligase (Parkin, ab15954) and total oxidative phosphorylation (OXPHOS, ab110413) rodent western blot (WB) antibody cocktail were purchased from Abcam (Cambridge, MA, USA). Antibodies against nuclear respiratory factor 1 (NRF1, sc-33771), voltage dependence anion channel (VDAC, sc-8828), phosphatase and tensin homologue (PTEN) induced kinase 1 (PINK1, sc-33796), transcription factor A, mitochondria (TFAM, sc-19050) were purchased from Santa Cruz Biotechnology (Dallas, TX, USA). Antibody against mitofusin 2 (MFN2, D2D10) was purchased from Cell Signaling Technology (Danvers, MA, USA). All the other reagents were analytical grade. 

### 2.2. Cell Culture and Cell Viability

The LLC-PK1 cells were cultured in DMEM medium, supplemented with 10% FBS and penicillin/streptomycin (100/50 U/mL) in a humidified incubator with 5% CO_2_ atmosphere at 37 °C. The experiments were carried out at a density of 50,000 cell/cm^2^, passaged 9 to 30 times since the acquired cryovial was opened. The cells were grown in 100 mm culture dishes and when they reached 90% confluence, were planted in the corresponding experimental plates. After 24 h of growth in 10% FBS medium, LLC-PK1 cells were exposed to 0 to 5 μM αM, 5 to 40 μM CDDP or co-incubation of αM and CDDP for 24 h in 1% FBS medium. Stock 10 mM αM was dissolved in DMSO and a posterior dilution of 1:100 in phosphate buffer saline (PBS) (100 μM) was used to prepare each experimental concentration. A stock 0.5 mg/mL (1.670 mM) of CDDP in 0.9% saline solution was used for different CDDP concentrations. Additional treatment consisted of pre-treatment with 30 μM CQ (2 h) or 10 nM wortmannin (1 h previous and during different treatment: αM, CDDP or αM+CDDP) as autophagy inhibitors. After pre-treatment, αM and/or CDDP were added for 24 h. One bright field image was captured in triplicate with a 20× objective after treatments, using a Cytation 5 Cell Imaging Multi-Mode reader (BioTek Instruments, Winooski, VT, USA). 

Cell viability was measured by the FDA assay according to a previously described method [26] in 96-well tissue culture microplates, where fluorescence is directly proportional to cell viability.

### 2.3. Cell Respirometry

The oxygen consumption experiments in intact cells were performed using a high-resolution respirometry equipment O2k meter (Oroboros Instruments, Innsbruck, Austria) according to a method previously described [27]. Cells were pretreated with the corresponding schemes of αM, CDDP or co-incubation for 24 h. Subsequently, cells were washed with PBS, harvested with trypsin, and quantified by the trypan blue assay. Determinations were made using 2 mL of culture medium with 10% FBS. Each experiment was initiated by the addition of approximately 2.5 million cells, the respiratory parameters were defined as: 1. Routine respiration, corresponding to oxygen consumption with presence only of cells. 2. Leak, corresponding to cellular oxygen consumption in the presence of 5 μM oligomycin. 3. Maximum capacity of electron transfer system (E) was achieved by titrations with 5 μM DNP. 4. Respiratory control (RC) corresponding to the ratio basal/leak. 5. Respiration associated with oxidative phosphorylation (P) was calculated by the formula Routine-Leak. All parameters were corrected by subtracting the non-mitochondrial respiration, obtained by the addition of 1 μM rotenone plus 5 μM antimycin A, and normalized by the number of cells.

Complex I (CI)-linked respiration was measured in a different experiment by the rest of routine respiration minus the respiration after inhibition with 1 μM rotenone. The activity of complex IV (CIV) was evaluated in intact cells in culture medium with 10% FBS supplemented with 0.5 µM rotenone plus 2.5 µM antimycin A. Oxygen consumption was stimulated by the addition of 0.5 mM TMPD plus 2 mM ascorbate. CIV activity was corrected by oxygen consumption in the presence of the appropriate inhibitor (100 mM NaN_3_). The results were normalized by the number of cells.

### 2.4. Total Protein Extract and Western Blot

LLC-PK1 cells were grown on 60 mm plates, after treatment were washed twice with cold PBS and lysed by addition of 400 μL of cold radioimmunoprecipitation assay buffer (RIPA) buffer (50 mM Tris-HCl, pH 7.4, 150 mM NaCl, 1 mM EDTA, 0.5% sodium deoxycholate, 1% NP-40, 0.1% SDS) with protease and phosphatase inhibitors (25 mM NaF, 1 mM Na_3_VO_4_, 1 mM PMSF, 1 mM Na_4_P_2_O_7_, 0.5 mM glycerophosphate and 1X protease inhibitors cocktail) for 30 min at 4 °C with stirring. Lysates were centrifuged 15,000 × *g* during 10 min at 4 °C and the supernatant was collected and stored at −70 °C until the experiment was carried out. The protein concentration was quantified by the Lowry assay [28]. Equal amounts of protein (15 μg) were separated on 10% to 15% in sodium dodecyl sulfate–polyacrylamide gel electrophoresis (SDS-PAGE) as appropriate. Proteins were transferred onto Immobilion PVDF membranes for fluorescent application on wet transfer and blocked with 5% non-fat dry milk for 1 h. Then incubated overnight at 4 °C with the appropriate primary antibody: MDA (1:2000), 4HNE (1:3000), PGC-1α (1:100), NRF1 (1:200), TFAM (1:500), VDAC (1:500) OXPHOS cocktail (1:1500), MNF2 (1:1000), PINK1 (1:1000), Parkin (1:1000), LC3 (1:1500), p62 (1:1000) and α-Tub (1:8000). Then the membranes were incubated with the corresponding fluorescent secondary antibodies (1:10000) for 1 h at room temperature in darkness. Protein bands were detected in an Odyssey scanner (Li-COR Biosciences, Lincoln, NE, USA) and protein band intensity was analyzed by Image StudioTM Life Software Li-COR Odyssey, which led us to normalize to correct for loading variations between bands and give us signal counts measured in a single pixel per unit time through Z-Factor Calculation. In all cases, except OXPHOS subunits, α-TUB served as loading control. Thus, once the protein target was detected, we proceeded to detect its loading control. For that, the membranes were rinsed only with Tris-buffered saline, 0.1% Tween 20 (TBS-T 1x) 3 times for 5 min and then the membranes were incubated with primary α-TUB antibody, following the usual steps as for protein target. The OXPHOS subunits were referred to reversible Ponceau staining as loading control as described by Romero-Calvo et al. [29]. Briefly, after transfer, the membranes were incubated in 1% Ponceau S solution for 2 min. Immediately after, membranes were rinsed with PBS to remove staining saturation, clearly visible bands. Membranes were placed between transparent sheets and scanned in conventional equipment at 300 dpi as TIFF document (HP Scanjet G4050). Again, membranes were rinsed until the staining completely disappeared. Finally, membranes were blocked with 5% non-fatty milk in TBS buffer, and the usual steps were continued. 

### 2.5. Mitochondrial Mass by MitoTracker Green

The mitochondrial mass was indirectly evaluated by a fluorometric assay using MitoTracker green (MTG). After treatment, cells were washed with DMEM w/o FBS and then incubated with 0.5 μM MTG for 20 min at 37 °C. After that, the cells were washed two times with mammalian Ringer Krebs Medium. Micrographs were taken with a 20× objective size with numerical aperture 0.45, and zoomed in with Cytation 5 imaging, GFP filter (Ex/Em, 469 nm/525 nm).

### 2.6. Statistical Analysis

Data are presented as the mean±standard deviation (SD). Data were analyzed by one-way ANOVA followed by the corresponding Dunnett or Tukey post-test. Comparison between time course and protein level as independent variables of all treatment were performed using two-way ANOVA followed by Bonferroni post-test using the software Graph-Pad Prism 5 (San Diego, CA, USA). A *p*-value lower than 0.05 was considered to deduce representative changes of the behavior studied with respect to each treatment.

## 3. Results

### 3.1. αM Prevented CDDP-Induced Cell Death

**Figure 1:**
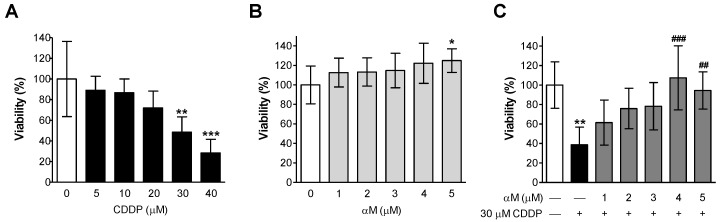
Protective effect of alpha mangostin (αM) against cis-dichlorodiammineplatinum II (CDDP) induced cell death. Lilly laboratory culture porcine kidney (LLC-PK1) cells were treated with (**A**) 0–40 μM cisplatin, (**B**) 0–5 μM αM or (**C**) co incubated with 30 μM CDDP and increasing concentrations of αM for 24 h. After treatment, cell viability was measured by the fluorescein diacetate (FDA) assay. The data are presented as mean ± SD, *n* = 5–7. *** *p* < 0.001, ** *p* < 0.01 and * *p* < 0.05 vs. control (0). ^###^ *p* < 0.001 and ^##^ *p* < 0.01 vs. CDDP.

LLC-PK1 cells were incubated with various CDDP concentrations: 5, 10, 20, 30, and 40 μM (Figure 1A) and it was observed that at 30 μM CDDP, reduced approximately 50% of cell viability, as we previously reported [26]. Later, cells were incubated with αM concentrations between 1 and 5 μM. As we expected, αM was not toxic up to 5 μM (Figure 1B). To evaluate protection by αM, αM and 30 μM CDDP were co-incubated. We observed that αM protected the cells against CDDP-induced cytotoxicity in a concentration-dependent manner and the protection with the least concentration was found at 4 μM (Figure 1C). So, the subsequent experiments were carried out with 4 μM αM and 30 μM CDDP.

**Figure 2:**
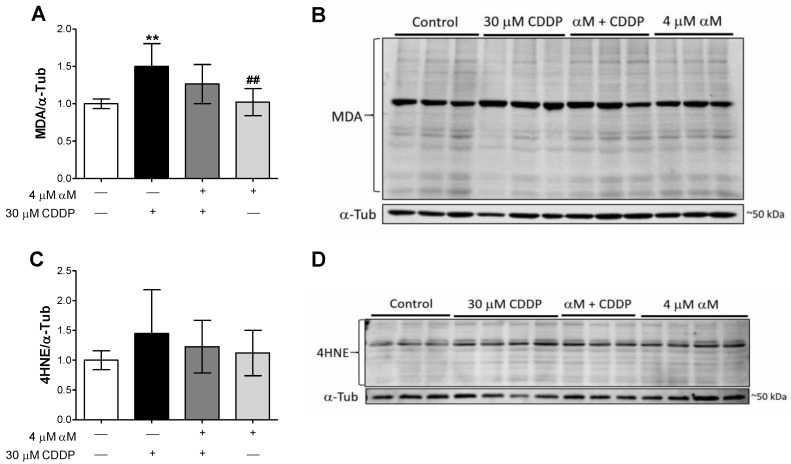
Changes in oxidative stress markers in LLC-PK1 cells treated with αM, CDDP or both. After 24 h of αM-, CDDP-treatment or both, the oxidative stress was evaluated by the lipoperoxidative markers (**A**) malondialdehyde (MDA) and (**C**) 4-hydroxynonenal (4HNE). (**A**) and (**C**) show the densitometry data and (**B**) and (**C**) show the representative blots. The data are presented as mean ± SD, *n* = 6–7. ** *p* < 0.01 vs. control. ^##^ *p* < 0.01 vs. CDDP. α-Tub = alpha-tubulin.

CDDP’s toxicity is partially associated with oxidative stress. To evaluate the effect of CDDP-induced oxidative stress, we evaluated the lipid peroxidation markers 4HNE and MDA by Western blot (WB). As we show in Figure 2, CDDP increased the level of MDA (Figure 2A,B) but not those of 4HNE (Figure 2C,D). αM was unable to prevent CDDP-induced MDA increase. 

### 3.2. αM’s Protection is Associated to the Preservation of Mitochondrial Bioenergetics

**Figure 3:**
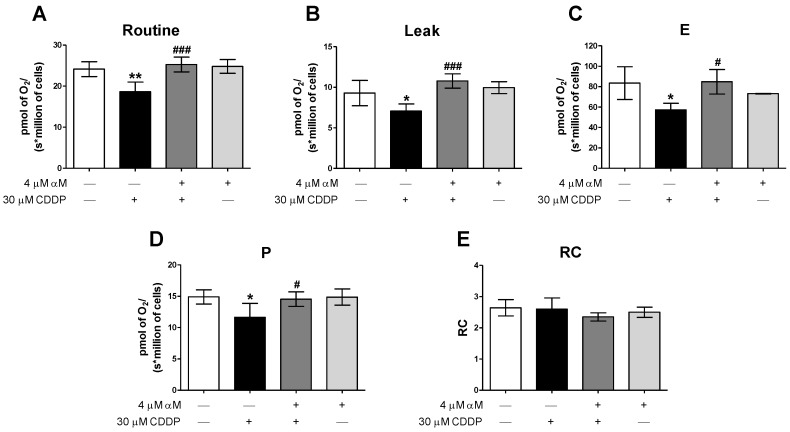
αM co-treatment prevents CDDP induced a decrease in respiratory parameters at 24 h. (**A**) Cellular routine respiration = Routine, (**B**) leak of respiration, (**C**) maximum capacity of electron transfer system = E, (**D**) respiration associated to oxidative phosphorylation = P, (**E**) respiratory control = RC. The data are presented as mean ± SD, *n* = 3–5. ** *p* < 0.01 and * *p* < 0.05 vs. control. ^###^ *p* < 0.001 and ^#^ *p* < 0.05 vs. CDDP.

The main nephrotoxic mechanism of CDDP is mitochondrial damage [13,14]. Therefore, we determined if the preservation of mitochondrial bioenergetics was related to protection by αM. So, we evaluated the respiratory states in the whole cells. As shown in Figure 3, CDDP-treatment reduced the Routine respiration, the Leak of respiration, the maximum capacity of the electron transfer system (E) and the respiration associated to oxidative phosphorylation (P) at 24 h. Interestingly, due to both Routine and Leak respiration reduced, the respiratory control (RC) did not change with CDDP-treatment (Figure 3E). All respiratory state alterations were reversed by αM co-treatment. Importantly αM alone did not alter any respiratory parameters at this concentration (Figure 3). 

**Figure 4:**
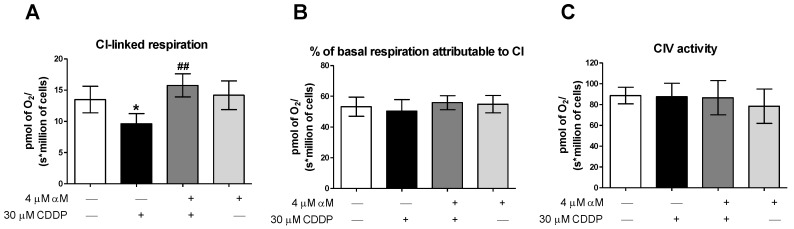
Determination of respiratory parameters at 24 h in LLC-PK1 cells treated with αM, CDDP or both. (**A**) Complex I (CI)-linked respiration, (**B**) percentage of respiration attributable to CI and (**C**) activity of complex IV (CIV). The data are presented as mean ± SD, *n* = 4–6. * *p* < 0.05 vs. control. ^##^ *p* < 0.01 vs. CDDP.

The decrease in all the respiratory states without changes in RC, suggested that the mitochondrial bioenergetics alterations were related to a reduction in the activity of the whole electron transport system (ETS). So, we evaluated the CI-linked respiration, the CIV activity and the percentage of routine respiration attributable to CI. The CDDP-treatment diminished CI-linked respiration but had no effect on CIV activity and the percentage of basal respiration attributable to CI was not reduced (Figure 4). These results, together with the decrease in E, imply a reduction in whole ETS activity induced by CDDP, which could be caused by a reduction in mitochondrial mass. 

### 3.3. Mitochondrial Respirometry Alterations are Associated to Mitochondrial Mass Decrease Related to Mitochondrial Biogenesis Reduction

**Figure 5:**
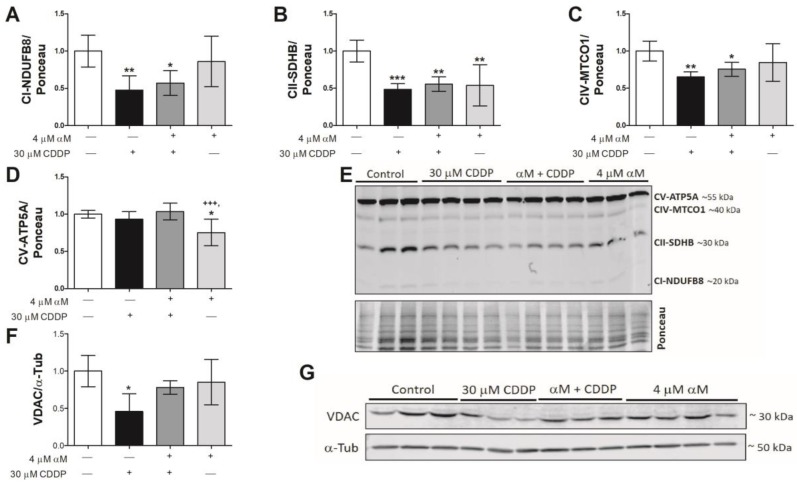
CDDP-induced toxicity is related to a decrease in mitochondrial proteins. LLC-PK1 cells were treated with αM, CDDP or both for 24 h. After treatment, western blotting of total oxidative phosphorylation (OXPHOS) cocktail in the whole cell (**A**–**D**,**F**) voltage-dependent anion channel (VDAC) as mitochondrial mass markers were quantified and (**E**,**G**) representative protein expression is shown. Densitometry values were normalized by Ponceau red staining. The data are presented as mean ± SD, *n* = 4–8. *** *p* < 0.001, ** *p* < 0.01 and * *p* < 0.05 vs. control. ^+++^ *p* < 0.001 vs. (αM + CDDP). NDUFB8 = NADH: ubiquinone oxidoreductase subunit B8, SDHB = succinate dehydrogenase complex iron–sulfur subunit B, MTCO1 = mitochondrial cytochrome c oxidase I catalytic subunit, ATP5A = ATP synthase F subunit alpha, α-Tub = alpha-tubulin.

**Figure 6:**
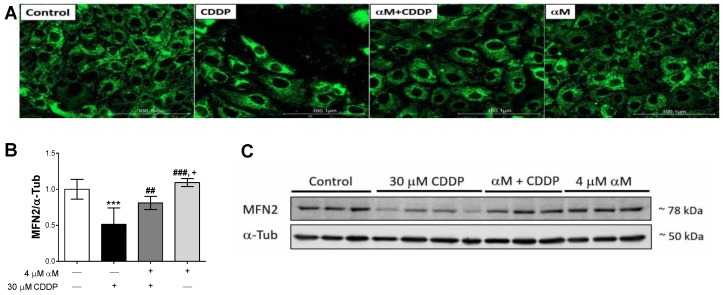
CDDP-induced toxicity is related to a decrease of mitochondrial fusion. LLC-PK1 cells were treated with αM, CDDP or both for 24 h. After treatment, (**A**) representative micrographs of MitoTracker green, mitochondrial mass marker, at 20x and posterior zoom 1:1 and western blotting of (**B**) Fusion marker, mitofusin 2 (MNF2) level and representative blot in (**C**). The data are presented as mean ± SD, *n* = 6–8. *** *p* < 0.001 vs. control. ^###^ *p* < 0.001, ^##^ *p* < 0.01 vs. CDDP. ^+^ *p* < 0.05 vs. (αM+CDDP). α-Tub = alpha-tubulin.

To determine if the reduction in the respiration states was linked to a decrease in mitochondrial mass, the protein levels of VDAC and some mitochondrial complex subunits were evaluated by WB. We evaluated mitochondrial subunits using an cocktail contained antibodies to labile mitochondrial subunits. So, they allow to evaluate the changes in the mitochondrial proteins of the ETS in the membrane only. Furthermore, several models of acute and chronic kidney damage have previously reported a reduction in protein levels of these subunits as well as in levels of their messengers, which has been related to a reduction in mitochondrial biogenesis and bioenergetics in such models [30,31,32,33,34,35]. Figure 5 shows that CI-NDUFB8 (Figure 5A), CII-SDHB (Figure 5B) and CIV-MTCO1 (Figure 5C) subunits were decreased with CDDP-treatment, whereas the CV-ATP5A subunit was unchanged (Figure 5D). Moreover, we found a decrease in VDAC protein levels with CDDP-treatment (Figure 5F,G), which was partially prevented by αM-treatment. To corroborate these results, we obtained representative images of the mitochondrial mass using MTG, a selective mitochondrial fluorescent label [36] and also MFN2, fusion marker. Figure 6A shows the images of CDDP treated cells, where a fragmented mitochondrial network was observed, with loss of continuity and fluorescence focal points. In addition, we found the MNF2 level in CDDP treatment that was avoided by αM co-treatment suggests fusion diminishing (Figure 6B,C). Together, these results support the idea that CDDP increases mitochondrial mass reduction and fragmentation, while αM can partially preserve it.

**Figure 7:**
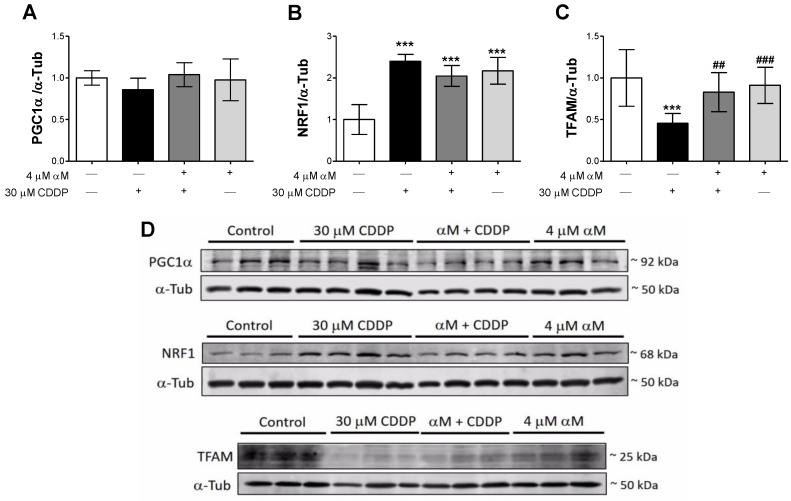
Modulation of mitochondrial biogenesis in LLC-PK1 cells treated with αM, CDDP or both. After 24 h of treatment, protein levels of (**A**) peroxisome proliferator-activated receptor gamma (PPARγ) coactivator 1-alpha (PGC1α), (**B**) nuclear respiratory factor 1 (NRF1), (**C**) mitochondrial transcription factor A (TFAM) were quantified. Representative blots are shown in (**D**). The data are presented as mean ± SD, *n* = 5–7. *** *p* < 0.001. ^###^ *p* < 0.001 and ^##^ *p* < 0.01 vs. CDDP. α-Tub = alpha-tubulin.

To determine if the observed reduction in mitochondrial mass was related to alterations in mitochondrial biogenesis, we evaluated the levels of mitochondrial biogenesis proteins PCG-1α, NRF1, and TFAM. Interestingly NRF1 levels were upregulated in all treatments (Figure 7B), although we expected a reduction. The implications of these changes are deliberated in the discussion section. Furthermore, although we did not observe changes in PGC-1α (Figure 7A) CDDP-treatment downregulated TFAM levels (Figure 7C) and αM co-treatment prevented it. These results suggest that mitochondrial mass reduction is partially attributable to the decrease in mitochondrial biogenesis induced by CDDP. 

### 3.4. The Induction of Mitophagy is Related to Protection by αM

**Figure 8:**
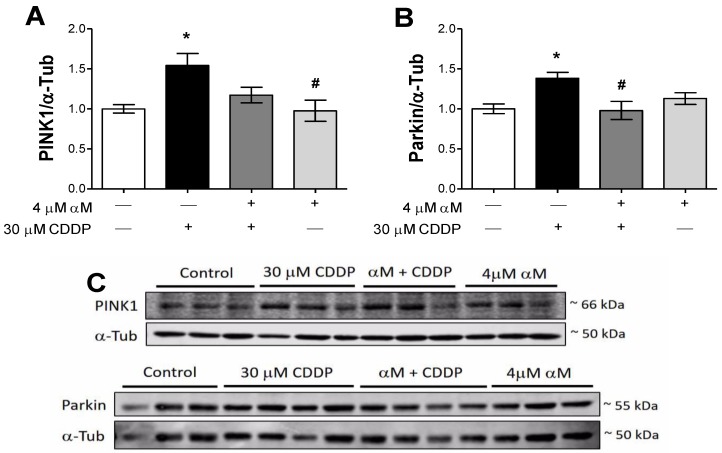
Modulation of mitophagy induction in LLC-PK1 cells treated with αM, CDDP or both. After 24 h of αM-, CDDP-treatment or both, mitophagy proteins (**A**) phosphatase and tensin homologue (PTEN)-induced kinase 1 (PINK1) and (**B**) parkin RBR E3 ubiquitin protein ligase (Parkin) were evaluated. Representative blots are shown in (**C**). The data are presented as mean ± SD, *n* = 6–7. * *p* < 0.05 vs. control. ^#^ *p* < 0.05 vs. CDDP. α-Tub = alpha-tubulin.

**Figure 9:**
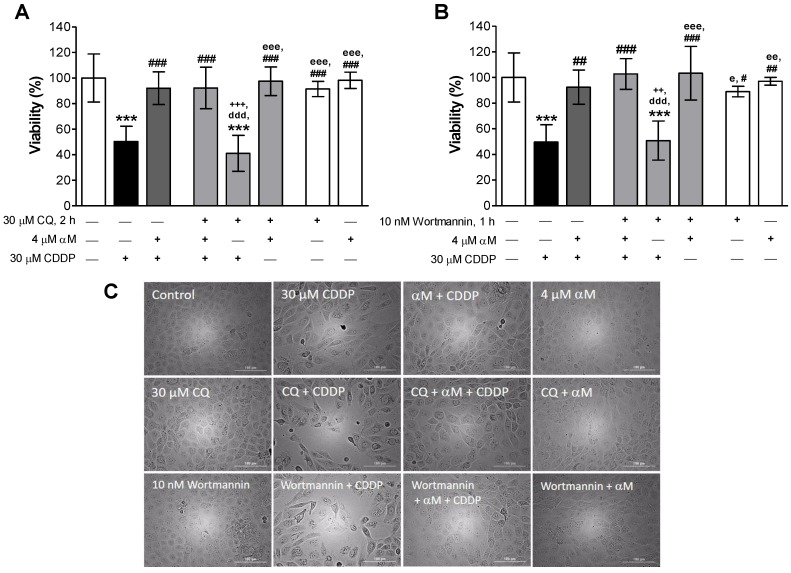
Effect of autophagy inhibition on the protection induced by αM vs. CDDP. LLC-PK1 cells were pretreated with (**A**) 30 μM chloroquine (CQ) for 2 h, as an inhibitor of degradation of cargo in the final autophagy phase, or (**B**) 10 nM wortmannin for 1 h and during the experiment, as an inhibitor of phosphoinositide 3-kinase (PI3K) in the initial autophagy phase induction. After pre-treatment, the cells were co-incubated with 4 μM αM and/or 30 μM CDDP for 24 h. Cell viability was determined, and (**C**) representative micrographs were taken. The data are presented as mean ± SD, *n* = 4–6. *** *p* < 0.001 vs. control. ^###^ *p* < 0.001, ^##^ *p* < 0.01 and ^#^ *p* < 0.05 vs. cisplatin. +++ *p* < 0.001 and ++*p* < 0.01 vs. (αM+CDDP). ^ddd^ *p* < 0.001 vs. (stressor+αM+CDDP). ^eee^ *p* < 0.001, ^ee^ *p*<0.01 and ^e^ *p* < 0.05 vs. (stressor+CDDP).

Dysfunctional mitochondria are usually eliminated through diverse processes, such as mitophagy [13], so we evaluated if mitophagy was related to CDDP-induced decrease in mitochondrial mass. Figure 8 shows that CDDP increased both PINK1 (Figure 8A) and Parkin (Figure 8B) protein levels and this increase was prevented by αM co-incubation. As the mitophagy process can be related to general macroautophagy, we pre-incubated LLC-PK1 cells with 30 μM CQ for 2 h before αM or CDDP-treatment, and with 10 nM of wortmannin 1 h before and during treatment, as autophagy inhibitors (Figure 9). Although autophagy inhibition has been related to the worsening of CDDP injury, Figure 9A shows that, apparently, CQ only has an effect on CDDP-treatment and does not have any effect over the protection by αM. CQ pre-treatment did not reduce cell viability in αM+CDDP-treatment. Wortmannin, as well as CQ, did not have any effect over the protection by αM (Figure 9B). Probably protection by αM has a mechanism independent of the induction of autophagy. Figure 9C shows micrographs representative of all treatments. CDDP treated cells showed less density at 24 h, as well as cell swelling and loss of structure compared to the control group. αM co-treatment prevented CDDP-alterations. In addition, in CQ and wortmannin + CDDP. we observed similar damage to the CDDP group. However, CQ or wortmannin did not alter the morphological characteristic compared with the αM+CDDP treatment. 

**Figure 10:**
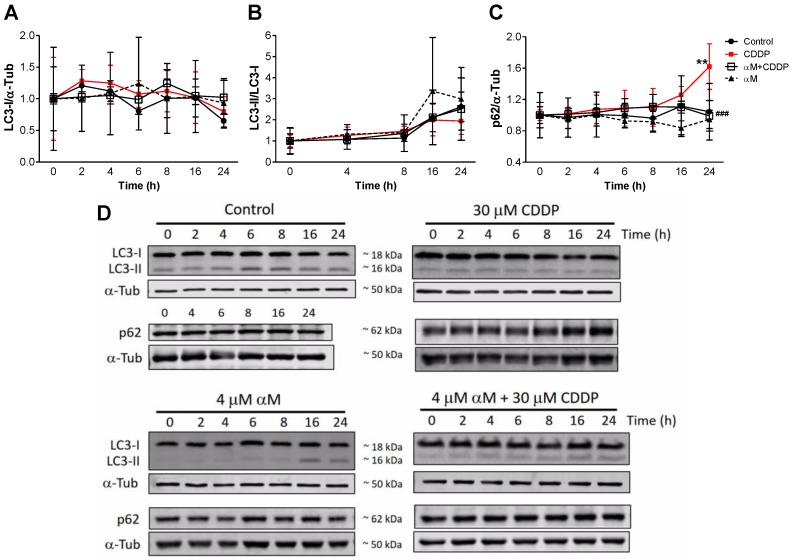
Time-course effect of αM and CDDP on autophagy stress markers. LLC-PK1 cells were incubated with 4 μM αM, 30 μM CDDP or both during 0, 2, 4, 6, 8, 16, and 24 h. After each treatment, (**A**) microtubule-associated protein 1 light chain 3 alpha I (LC3-I), (**B**) lipidated form of LC3 (LC3-II), and (**C**) ubiquitin-binding protein p62 (p62) protein levels were evaluated. Panels A, B, and C show quantitative data, and panel D shows representative blots. Data are presented as mean ± SD, *n* = 3–5. ** *p* < 0.01 vs. control and ^###^ *p* < 0.01 vs. CDDP. α-Tub = alpha-tubulin.

Figure 10C shows that CDDP-treatment induced an increase in p62 levels at 24 h, and a slight increase in microtubule-associated protein 1 light chain 3 alpha I (LC3-I) (Figure 10A) and the LC3-II/LC3-I ratio (Figure 10B). The increase in p62 is relevant because of the fact that p62 has been reported as one of receptors involved in PINK1/Parkin-mediated mitophagy balance [37] and aberrant p62 levels may be the result of mitochondrial dysfunction [38], which together with the autophagy results (Figure 9), suggest that mitophagy is involved in the observed mitochondrial mass decrease induced by CDDP.

## 4. Discussion

Cisplatin (CDDP) is highly deleterious at proximal tubule level given its high density of mitochondria because of its reabsorption function [13]. Its nephrotoxicity is attributable to high CCDP accumulation in the kidneys and adverse impacts on the renal transport system. CDDP entry into the tubular cells by passive diffusion or by a number of cellular transporters including human copper transport protein 1 (Ctr1) and the organic cation transport 2 (OCT2), which are highly expressed on renal tubular cells [39]. Although this transport is expressed in other tissue, diverse strategies have demonstrated its importance in nephrotoxicity development [2]. Inside cells, CDDP metabolism results in its activation to a more potent toxin and glutathione-cisplatin-conjugate derivate and compromises glutathione level and synthesis [40]. CDDP can bind to proteins of cytosol, mitochondria, and ER [2,40,41]. Furthermore, CDDP binding to nuclear and especially mitochondrial DNA, trigger mitochondrial dysfunction [2]. Although hydration and diuretics are used as a strategy to prevent CDDP-induced nephrotoxicity, the optimal strategy to prevent this pathology is still being sought [1]. For this reason, new strategies are being developed that include using phytochemical compounds such αM [5]. 

αM has many biological functions [6,8]. There are three studies that demonstrated nephroprotection versus CDDP, without interfering with urinary secretion or antiproliferating activity [3,7,9]. Alpha-mangostin is not specific to tubular cells; however, it preserves the renal function. Pharmacokinetics studies have shown that it can be quantified in the kidney and liver [6] as well as its nephroprotective effects [3,7,9,11]. αM protection in CDDP nephrotoxicity related to its antioxidant properties has been described [3,7,9]. To study αM mechanism we used the LLC-PK1 cell line because it is very stable, undergoing little to no transformation or neoplastic change after numerous passages [42] and it shows tubular cells characteristics [43,44]. We corroborated in LLC-PK1 cells that αM co-incubation protects against CDDP-induced (30 μM) viability decrease (Figure 1C). The protection was found at 4 μM and 5 μM, however, no differences were found between 4 and 5 μM (Figure 1), this time using a much purer αM extract than the previous report [9,25]. It has been previously reported by 3-(4,5-dimethylthiazol-2-yl)-2,5-diphenyltetrazolium bromide (MTT) viability assay 7.5 μM αM reduced viability cell [10], we reproduced this experiments with 1, 2.5, 5, 7.5, and 10 μM and found that αM can reduce near 50% viability at 7.5 μM (data not show). For this reason, we decide to use the minimum αM concentration in the present study. Our group has previously demonstrated that CDDP induces oxidative stress in vitro, and αM was able to prevent the increase in ROS and the decrease in glutathione (GSH) [9]. In our study, αM was unable to prevent MDA increase (Figure 2A). We are tempted to speculate that the use of different markers (MDA versus ROS and GSH) may explain the differences in both studies. 

In this study, we demonstrated that CDDP induced mitochondrial bioenergetics alterations, characterized by the reduction in the respiratory parameters: routine, leak, E, and P (Figure 3), as well as CI-linked respiration (Figure 4). The absence of change in RC (Figure 3E), suggests that the CDDP-induced respiratory state alterations were related to a decrease in whole ETS activity rather than a reduction in a specific complex. Which was congruent with the absence of a reduction in the percentage of basal respiration attributable to CI (Figure 4B) and would be responsible for the decrease in OXHPOS capacity (Figure 3C). It was also demonstrated for the first time that αM prevents the bioenergetics alterations induced by CDDP as we can see by the preservation of all evaluated mitochondrial parameters in the co-treatment group (Figure 3 and Figure 4). Mitochondrial dysfunction has been widely recognized as an important factor in renal disease, especially in tubular damage [13]. Furthermore, in the CDDP model, the oxidative stress and the mitochondrial dysfunction favor the activation of apoptosis, which contributes to renal damage [15,17,45]. So, the protection by αM would be, at least in part, attributable to mitochondrial preservation (Figure 3 and Figure 4), which explains the protection of tubular epithelial cells.

Furthermore, the decrease in the levels of VDAC and respiratory complex subunits (Figure 5A–F), implies that the observed mitochondrial bioenergetics alterations are attributable to mitochondrial mass reduction and may also be attributable to mitochondrial network fragmentation. This is congruent with a previous report that demonstrates a shift of mitochondrial dynamics towards fission in CDDP-induced nephropathy [14]. Although the co-treatment with αM had no effect in the evaluation of mitochondrial protein subunit levels (Figure 5A–F), it prevented the alteration in the morphology of the mitochondrial network (Figure 5G), which is congruent with the preservation of mitochondrial dynamics, demonstrated by other antioxidants, such as curcumin in CDDP-induced damage nephropathy [14]. 

Our results also suggest that the observed reduction in mitochondrial mass is attributable to decreased mitochondrial biogenesis. We found a decrease in TFAM levels in CDDP-treated cells (Figure 6C,D), but PGC1α levels did not change (Figure 6A), suggesting a reduction in mitochondrial biogenesis. We suggest this, first, because although PGC-1α expression did not change, its activity also has to be considered. PGC-1α is regulated via phosphorylation by protein kinase B (AKT) and adenosine monophosphate-activated protein kinase (AMPK), as well as deacetylation by sirtuins [46]. In fact, it has been reported by Ortega-Dominguez [14] that CDDP decreased sirtuin-3 the levels, which triggers the increase in lysine acetylated levels. Therefore, this evidence supports the idea that PGC-1α is inactive in the cisplatin group. Second, as with PGC-1α, NRF1 expression does not necessarily correlate with its activity. NRF1 activity can be regulated by phosphorylation and/or by interactions with PGC-1α, PGC-1β, and PRC [14,46]. In this sense, TFAM levels determination is an indirect proof of its activity. About it, CDDP exacerbated NRF1 levels, whereas TFAM decreased. So, it can be suggested that CDDP-induced NRF1 overexpression is not functional, unlike the co-incubation with αM. Taken together, we can suggest cisplatin suppress the mitochondrial biogenesis pathway. Interestingly, we found increased levels of NRF1 in CDDP-treated cells (Figure 6B). This induction can be explained by the fact that the promoter of the NRF-1 gene possesses antioxidant response elements. Therefore, the nuclear factor erythroid 2-like 2 (Nrf2) can induce its expression [47]. Although we did not evaluate Nrf2 induction in this work, our group has previously reported that CDDP induces the increase in heme oxygenase-1 (HO-1) levels in LLC-PK1 [26], leading to Nrf2 activation, so the observed NRF1 increase can be the consequence of an adaptive response of Nrf2 activation. On the other hand, it was documented that αM can induce Nrf2 activation in retinal cells and liver tissue [48,49], which can also explain the observed increase in NRF1 levels in co-treatment and αM groups (Figure 6B,D), but more experiments are necessary to demonstrate Nrf2 induction.

In view of the fact that mitochondrial mass is regulated by biogenesis and mitophagy [13], we evaluated the mitophagy markers Pink1/Parkin. Co-incubation with αM avoided Parkin (Figure 7B) and PINK1 (Figure 7A) increase induced by CDDP. Recently, mitophagy was described as a response mechanism in CDDP-induced nephrotoxicity, and the PINK/Parkin pathway was suggested as the main route to remove damaged mitochondria [22,23,24]. The mitophagy machinery can be shared with the macroautophagy, but can also be independently-activated [19]. We show that pharmacological autophagy inhibition with CQ or with wortmannin did not have any effect in the protection by αM and did not increase CDDP toxicity (Figure 8). However, the increase in p62 levels (Figure 9C,D) and the no change of the LC3 ratio in CDDP-treatment at 24 h suggest that increases of PINK/Parkin can use the p62 protein as an adaptor, as suggested in a previous report [37]. However, it is also known that the accumulation of misfolded proteins leads to aberrant p62 expression [38]. Altogether, our results support the idea of a partial preservation of mitochondrial mass and function by αM-treatment. 

We found 4 μM of αM did not change the respiratory parameters (Figure 3 and Figure 4) nor the expression of mitochondrial proteins (Figure 5), except CII-SDHB (Figure 5B) and CIV-MTCO1 (Figure 5C). Unfortunately, nowadays, we do not have enough information to explain the origin of these changes. However, it is important to note, that although these subunits decreased in αM treatment, the functional respirometry studies did not show changes in any of the evaluated parameters with respect to the control group (Figure 3 and Figure 4). This implies the observed CII-SDHB and CV-ATP5A decrease did not change the mitochondrial bioenergetics in the αM treatment with respect to the control. And we did not observe changes in the mitochondrial network by MTG (Figure 6A). Additionally, αM alone did not induce mitochondrial biogenesis (Figure 7) or mitophagy activation (Figure 8), implying that at least at this concentration, αM has no negative effects on mitochondrial function. In support of this, studies in heart tissue (200 mg/kg) and hepatic cells (30 μM) describe that αM can avoid mitochondrial dysfunction (preventing the decrease in activity and levels of antioxidant proteins and in RC, as well as ROS increase) induced by isoproterenol or free fatty acids increase [50,51]. By contrast, reports also describe that high αM concentrations (above 25 μM) can induce mitochondrial alterations in mitochondria isolated from kidneys [52], indicating that αM effects are tissue- and concentration-dependent. Although there are no clues regarding αM induced proliferation in normal cells, we evaluated GRP94, an ER chaperon, with 4 μM αM and found can it induce proliferation at 24 h of incubation (data not show). GRP94 is a glycoprotein related to protein folding, stores of calcium and targeting proteins to ER-associated degradation (ERAD), the client protein of GRP94 is related to specialized functions in immunity, grown signaling, and cell adhesion [53]. Probably, GRP94 induction in αM treatment can be related to grown signaling and cell adhesion, and this can explain this result, but more experiments are necessary. 

**Figure 11:**
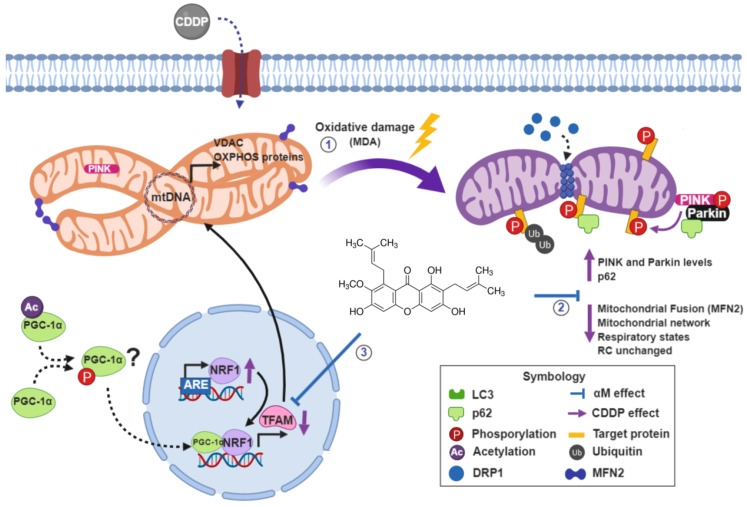
Integrative scheme. CDDP induces (1) the increase MDA level, (2) decrease respiratory states (except respiratory control, RC), the mitochondrial network fragmentation, the mitochondrial fusion protein 2 (MNF2) decrease and induce mitophagy (PINK1/Parkin and p62 increase). Furthermore, CDDP induces (3) TFAM reduction which reduces mitochondrial protein (VDAC and OXPHOS) and probably mitochondrial transcription, and also induces an NRF1 increase. The αM prevented all alterations, except NRF1 induction. Created with BioRender.com.

As we show in the integrative scheme (Figure 11) our results suggest that 30 μM CDDP-treatment induces alterations in LCC-PK1 cells principally by reducing mitochondrial function and mass, which is partially attributable to mitochondrial biogenesis reduction and mitophagy activation. αM can prevent mitochondrial bioenergetics alterations and the induction of mitophagy. These mechanisms would be involved in the protective effects observed in LLC-PK tubular cells.

## 5. Conclusions

αM’s protective effects in CDDP-induced toxicity in LLC-PK1 are mostly attributable to mitochondrial mass and function preservation.
